# Differential associations of traditional and shape-based adiposity indices with steatosis and fibrosis risk in metabolic dysfunction–associated steatotic liver disease

**DOI:** 10.1007/s40618-026-02908-1

**Published:** 2026-06-01

**Authors:** Giulio Geraci, Valentina Paternò, Alessandro Vitello, Marcello Maida, Federico Bonomo, Samuele Sanfilippo, Simone Argento, Carlo Maida, Giuliano Cassataro, Roberto Baratta, Gabriele Costanzo, Francesco Pallotti, Calogero Geraci, Roberta Esposito, Riccardo Polosa, Pietro Ferrara

**Affiliations:** 1 Department of Medicine and Surgery, “Kore” University of Enna, Enna, Italy; 2 Unit of Internal Medicine, “Umberto I” Hospital, Enna, Italy; 3 Gastroenterology Unit, “Umberto I” Hospital, Enna, Italy; 4 Unit of Internal Medicine, “S. Elia” Hospital, Caltanissetta, Italy; 5 Department of Medicine and Pulmonology, Fondazione Istituto “G. Giglio”, Cefalù, Italy; 6 Endocrinology Unit, ARNAS (Azienda di Rilievo Nazionale e di Alta Specializzazione – National High-Specialty Healthcare Trust) Garibaldi Hospital, Catania, Italy; 7 Endocrinology Unit, Azienda Sanitaria Provinciale of Catania (ASP Catania), Catania, Italy; 8 Cardiology Unit, Azienda Sanitaria Provinciale of Caltanissetta (ASP Caltanissetta), Caltanissetta, Italy; 9 Department of Clinical Medicine and Surgery, Federico II University Hospital, Naples, Italy; 10 Department of Clinical and Experimental Medicine, University of Catania, Catania, Italy; 11 Centre of Excellence for the Acceleration of HArm Reduction (CoEHAR), University of Catania, Catania, Italy; 12 Center for Public Health Research, University of Milan–Bicocca, Via Cadore 48 – U38 Building Monza I, Monza, 20900 Italy; 13 Laboratory of Public Health, IRCCS Istituto Auxologico Italiano, Milan, Italy

**Keywords:** Anthropometric indices, Hepatic steatosis, MASLD, Metabolic dysfunction, Obesity, Fibrosis stratification

## Abstract

**Background:**

Traditional anthropometric indices reflect total body adiposity, whereas novel shape-based indices capture fat distribution and unfavorable adiposity. How these measures relate to hepatic steatosis and fibrosis risk across the spectrum of metabolic dysfunction–associated steatotic liver disease (MASLD) remains unclear.

**Methods:**

In this cross-sectional study, 222 patients with MASLD were included. Body mass index (BMI), waist circumference (WC), a body shape index (ABSI) and body roundness index (BRI) were evaluated. Hepatic steatosis was assessed by ultrasonography using the Bright Liver Echo Pattern (BLEP), while fibrosis risk was estimated using the Fibrosis-4 (FIB-4) index.

**Results:**

BMI, WC, and BRI were higher in individuals with moderate-to-severe hepatic steatosis (BLEP ≥ 2), whereas ABSI did not differ according to steatosis severity. In multivariable logistic regression, BMI was independently associated with BLEP ≥ 2 (OR 1.19; 95% CI 1.05–1.35; *p* = 0.006), together with fasting glucose. ABSI showed a positive correlation with FIB-4 (*r* = 0.248; *p* < 0.001), whereas BMI did not. In multivariable linear regression, ABSI remained independently associated with FIB-4 (β = 0.145; *p* = 0.017), while BMI and BRI were not. Receiver operating characteristic analyses showed that BMI and WC best discriminated moderate-to-severe steatosis, whereas ABSI demonstrated the highest accuracy for identifying advanced fibrosis risk (FIB-4 ≥ 2.67; AUC 0.714; *p* = 0.004).

**Conclusions:**

Traditional and shape-based anthropometric indices capture complementary aspects of MASLD. Integrating measures of total adiposity and body shape may improve risk stratification in MASLD, although findings require confirmation using direct measures of liver fibrosis.

## Introduction

Obesity is a major driver of cardiovascular and metabolic diseases, and its prevalence is steadily increasing, currently affecting more than one billion people worldwide [1]. Among the numerous metabolic complications, metabolic dysfunction–associated steatotic liver disease (MASLD) is the most prevalent, affecting approximately 35% of the adult population [2, 3], and is strongly associated with an increased risk of cardiovascular disease and all-cause mortality [4, 5]. MASLD is defined by the presence of hepatic steatosis in individuals with one or more cardiometabolic risk factors, in the absence of significant alcohol consumption, and encompasses a disease spectrum ranging from isolated steatosis to metabolic dysfunction–associated steatohepatitis (MASH) and progressive liver fibrosis [6]. A large body of experimental and clinical evidence has demonstrated a close relationship between MASLD and total body adiposity [7]. However, both the development and progression of MASLD, as well as its cardiovascular burden, depend not only on the amount of adipose tissue but also on its regional distribution and metabolic activity [7, 8]. In particular, excess visceral adipose tissue has been consistently linked to higher cardiometabolic risk compared with subcutaneous fat, independently of the assessment technique used [9].

Traditionally, obesity has been assessed using simple anthropometric indices such as body mass index (BMI) and waist circumference (WC) [10]. However, these measures have important limitations, as they do not distinguish between fat and lean mass and provide limited information on adipose tissue distribution, a key determinant of clinical outcomes [10]. Moreover, traditional indices have shown inconsistent performance in predicting all-cause mortality across different populations [11, 12].

More recently, novel anthropometric indices, such as A Body Shape Index (ABSI) and Body Roundness Index (BRI), have been developed to better characterize body geometry and adipose tissue [12–14]. These indices have demonstrated stronger associations with all-cause mortality, cardiovascular risk, and subclinical organ damage compared with traditional measures, supporting their potential to identify metabolically adverse adiposity phenotypes [15–18].

Despite this growing body of evidence, the relationship between novel anthropometric indices reflecting fat distribution and the different hepatic manifestations of MASLD remains incompletely defined [19]. In particular, it is unclear whether these indices provide additional information beyond traditional measures of adiposity in capturing steatosis severity and fibrosis risk across the MASLD spectrum.

The aim of the present study was therefore to evaluate whether hepatic steatosis, assessed by ultrasound using the bright liver echo pattern (BLEP) [20, 21], and liver fibrosis risk, estimated using the well-validated non-invasive Fibrosis-4 (FIB-4) index score [22–24], are more closely associated with the novel adiposity indices ABSI and BRI than with traditional measures such as BMI and WC.

## Materials and methods

### Study population

This cross-sectional study included 222 Caucasian adult patients with hepatic steatosis and a MASLD diagnosis, consecutively attending the Umberto I Hospital in Enna for a comprehensive metabolic and hepatic evaluation between September 1, 2024, and November 1, 2025. Hepatic steatosis was a mandatory inclusion criterion and was assessed by ultrasonography using BLEP [20, 21]. All patients fulfilled the diagnostic criteria for MASLD according to current international guidelines [23, 24].

The exclusion criteria were as follows:

- Age < 18 years or > 80 years;
- Significant alcohol consumption (> 30 g/day in men and > 20 g/day in women) [25];
- Chronic liver diseases of other aetiologies, including hepatitis B or C, autoimmune, cholestatic, or known genetic liver diseases;
- Liver cirrhosis or hepatocellular carcinoma (HCC);
- Estimated glomerular filtration rate (eGFR) < 30 mL/min/1.73m^2^.
- Use of medications known to significantly affect lipid metabolism or body fat distribution (e.g., systemic corticosteroids, antiretroviral agents, or chemotherapeutic drugs), when deemed clinically relevant;
- Pregnancy or breastfeeding;
- Active malignancies, known systemic inflammatory conditions, or other major non-cardiovascular diseases.

Exclusion criteria were assessed through a detailed medical history, review of clinical records, and outpatient clinical evaluation. The study was approved by the local Ethics Committee and conducted in accordance with the Declaration of Helsinki.

### Study design

A complete medical history was obtained for all patients, together with a review of the available clinical documentation, and a standardized physical examination was performed. All individuals underwent anthropometric assessment for the calculation of adiposity indices and abdominal ultrasound examination. Fasting blood samples were taken to perform routine blood chemistry, and routine laboratory tests were measured using standardized methods with an automated analyzer (Boehringer Mannheim, Hitachi 911 system, Mannheim, Germany). The eGFR was calculated using the Chronic Kidney Disease Epidemiology Collaboration (CKD-EPI) equation [26].

### Anthropometric indices of adiposity

Body weight, height, and WC were measured by a physician according to standardized procedures. Body weight and height were taken with participants barefoot and wearing light clothing and were recorded to the nearest 0.1 kg and 0.1 cm, respectively. WC was measured with an inelastic tape to the nearest 0.1 cm at a midpoint between the lower margin of the rib cage and the upper border of the iliac crest, following gentle exhalation, as previously described [16–18]. BMI was calculated as the ratio between body weight (kg) and the square of height (m^2^). A Body Shape Index (ABSI), according to the formula proposed by Krakauer et al. [12], was calculated as:

$$\mathrm{ABSI}=\mathrm{WC}/(\mathrm{BMI}^{2/3} \times \mathrm{height}^{1/2}).$$

with WC expressed in meters. The BRI was derived from WC and height, both expressed in meters, through the preliminary calculation of eccentricity (ε), a parameter describing the degree of circularity of an ellipse, ranging from 0 (perfectly circular shape) to 1 (a vertical line), as previously described by Thomas et al. [14]:

$$\:\epsilon\:=\sqrt{1-\left(\frac{\left[\frac{WC}{2\pi\:}\right]^2}{\left[0.5\:\times\:height\right]^2}\right)}$$

We obtained the BRI formula, as follows:

$$\mathrm{BRI}=364.2-(365.5 \times \mathrm{e})$$

Higher BRI values reflect a more rounded body morphology, whereas lower values indicate a more elongated body shape.

### Ultrasound examination

Abdominal B-mode ultrasonography was performed using a GE Logiq P5 PRO instrument equipped with a low-frequency convex probe (3.5–5 MHz). All examinations were carried out by experienced operators who were blinded to the participants’ clinical and laboratory data. Ultrasound assessment was performed with the patient in the supine position after an overnight fast. Hepatic steatosis was defined according to the BLEP, based on increased echogenicity of the liver parenchyma compared with the kidney, attenuation of the ultrasound beam, reduced visualization of intrahepatic vascular structures, and reduced visualization of the diaphragm, as described elsewhere [20, 21, 23].

Hepatic steatosis was classified in a semi-quantitative manner as follows:

- BLEP 1: mild steatosis (grade 1);
- BLEP 2: moderate steatosis (grade 2);
- BLEP 3: severe steatosis (grade 3).

Mild steatosis was defined by a slight increase in hepatic echogenicity with preserved visualization of the diaphragm and intrahepatic vessels. Moderate steatosis was characterized by a more marked increase in echogenicity associated with partial attenuation of the ultrasound beam and reduced visibility of vascular structures. Severe steatosis was defined by marked hyper-echogenicity with significant beam attenuation and poor or absent visualization of deep hepatic structures and the diaphragm [21, 22, 27].

### Liver fibrosis risk

The risk of liver fibrosis was estimated using the FIB-4 index, a widely validated non-invasive score for the stratification of advanced fibrosis risk in chronic liver diseases, including MASLD [6, 22, 23]. FIB-4 was calculated according to the following formula: FIB-4 = (age [years] × AST [U/L])/(platelet count [10^9^/L] × √ALT [U/L]), where AST indicates aspartate aminotransferase and ALT alanine aminotransferase.

FIB-4 was used as a non-invasive tool for fibrosis risk stratification, consistent with its role in routine clinical practice. This approach reflects current recommended stepwise algorithms, in which FIB-4 is used as an initial risk stratification tool before second-line testing [22–24]. It was selected because of its simplicity, reproducibility, and high negative predictive value, making it particularly suitable as a first-line tool to exclude advanced liver fibrosis in both clinical and population-based settings. In accordance with major international guideline recommendations [23, 28], the following cut-off values were used for fibrosis risk stratification [22, 24]:

- FIB-4 ≤ 1.30: low risk of fibrosis;
- FIB-4 1.31–2.66: intermediate risk;
- FIB-4 ≥ 2.67: high risk of fibrosis.

### Statistical analysis

Continuous variables were reported as median and interquartile range (IQR), and categorical variables were summarized as frequency and percentage. Comparisons of variables were made by Mann–Whitney U test, Kruskal–Wallis test, Chi-square test and Fisher’s exact test as appropriate. A p-value of less than 0.05 was considered to indicate statistical significance. Logistic regression models were performed to identify the presence of variables independently associated with outcomes. Variables considered in the models were selected through stepwise model selection and guided by clinical relevance. To avoid the effect of collinearity, among anthropometric indices, traditional and shape-based adiposity measures were entered into separate regression models. Receiver operating characteristic (ROC) curve analyses were conducted to evaluate the ability of each adiposity index to discriminate individuals with BLEP ≥ 2 and FIB-4 ≥ 2.67. The area under the curve (AUC) was calculated for each index, and the statistical significance of the corresponding AUC was calculated for each index, and its statistical significance was assessed against the null hypothesis of no discrimination (AUC = 0.5). All statistical analyses were performed using SPSS v. 30.0 for Macintosh (SPSS Inc., Chicago, USA).

## Results

A total of 222 participants were included in the analysis. As shown in Table 1, individuals with moderate-to-severe hepatic steatosis (BLEP ≥ 2) had significantly higher body weight, BMI, WC, and BRI compared with those with mild steatosis, whereas ABSI did not differ between groups. In addition, the BLEP ≥ 2 group exhibited a less favorable metabolic profile, characterized by higher fasting glucose and lower HDL-cholesterol levels than individuals with BLEP < 2. No significant differences were observed between groups with respect to age, eGFR, diabetes, or FIB-4 values. Participant stratification according to FIB-4 categories is shown in Table 2. Traditional anthropometric measures, including body weight, BMI, and WC, did not differ significantly across FIB-4 classes, whereas both BRI and ABSI progressively increased with increasing FIB-4. The prevalence of individuals with BLEP ≥ 2 was also similar across FIB-4 classes.

**Table 1:** Demographic and clinical characteristics of the overall study population and stratified by hepatic steatosis severity (BLEP < 2 vs. ≥ 2)

	Overall study population (*n* = 222)	BLEP < 2 (*n* = 67)	BLEP ≥ 2 (*n* = 155)	*p*-value
Age (years)	46 (36–55)	48 (37–55)	46 (35–55)	NS
Males, *n* (%)	159 (71.6)	48 (71.6)	111 (71.6)	*NS*
Diabetics (%)	44 (19.8)	8 (11.9)	36 (23.2)	*NS*
Hypertensive (%)	54 (24.3)	15 (22.4)	39 (25.2)	*NS*
***Biochemical parameters***				
Serum glucose (mg/dL)	92.0 (83.0-107.0)	91.0 (81.0–97.0)	94.0 (84.0-110.0)	0.027
Serum uric acid (mg/dL)	5.8 (5.1–6.7)	5.7 (4.9–6.4)	5.9 (5.2–6.8)	*NS*
Serum creatinine (mg/dL)	0.84 (0.71–0.91)	0.87 (0.71–0.92)	0.81 (0.7–0.91)	*NS*
eGFR (mL/min/1.73m^2^)	104 (94–112)	102 (91–110)	104 (95–113)	*NS*
Serum total cholesterol (mg/dL)	201 (173–231)	204 (184–227)	198 (169–236)	*NS*
HDL-c (mg/dL)	47 (41–56)	49 (44–60)	46 (37–56)	0.008
Serum triglycerides (mg/dL)	134 (86–180)	120 (81–164)	137 (88–189)	*NS*
AST (U/L)	36.5 (28.0-49.5)	36.0 (26.0–42.0)	37.0 (29.0–54.0)	0.015
ALT (U/L)	65.0 (47.0–95.0)	52.0 (36.0–75.0)	71.0 (51.0-105.0)	< 0.001
Serum total bilirubin (mg/dL)	0.62 (0.46–0.86)	0.69 (0.48–0.86)	0.60 (0.46–0.86)	*NS*
Serum direct bilirubin (mg/dL)	0.20 (0.14–0.30)	0.21 (0.14–0.30)	0.19 (0.14–0.30)	*NS*
Serum albumin (g/dL)	4.7 (4.4–4.9)	4.6 (4.4-5.0)	4.7 (4.5–4.9)	*NS*
Platelet count (10^3^/µL)	219.0 (186.0-257.0)	219.5 (202.0-263.0)	218.5 (185.0-253.5)	*NS*
***Anthropometric parameters***				
Weight (Kg)	82.0 (70.9–88.9)	76.4 (68.0–85.0)	83.6 (75.5–93.0)	< 0.001
Height (cm)	168 (160–175)	169 (161–172)	168 (160–175)	*NS*
BMI (Kg/m^2^)	28.9 (26.2–32.0)	26.9 (24.8–30.1)	29.6 (26.6–32.8)	< 0.001
WC (cm)	100.00 (93.00-108.00)	96.00 (90.00-102.00)	102.00 (96.00-109.00)	< 0.001
BRI	5.53 (4.44–6.48)	4.73 (4.02–6.03)	5.69 (4.64–6.72)	0.004
ABSI	0.082 (0.079–0.085)	0.083 (0.079–0.085)	0.082 (0.079–0.085)	*NS*
***Liver scores***				
FIB-4	0.89 (0.65–1.39)	0.90 (0.63–1.39)	0.89 (0.65–1.41)	*NS*
FIB-4 > 1.3 (%)	61 (28.1)	20 (30.3)	41 (27.2)	*NS*
BLEP ≥ 2 (%)	155 (69.8)	0 (0)	155 (100)	< 0.001

BLEP: Bright liver echo pattern; eGFR: estimated Glomerular Filtration Rate; HDL-c: High Density Lipoprotein Cholesterol; AST: Aspartate aminotransferase; ALT: Alanine aminotransferase; BMI: Body Mass Index; WC: Waist Circumference; BRI: Body Roundness Index; ABSI: A Body Shape Index; FIB-4: Fibrosis 4 index score; NS: *p* > 0.05

**Table 2:** Demographic and clinical characteristics of study population stratified by FIB-4 categories

	FIB-4 ≤ 1.3 (*n* = 156)	1.3 < FIB-4 < 2.67 (*n* = 45)	FIB-4 ≥ 2.67 (*n* = 16)	*p*-value
Age (years)	39 (33–50)	56 (51–62)	62 (58–68)	< 0.001
Males, *n* (%)	118 (75.64)	28 (62.22)	9 (56.25)	*NS*
Diabetics (%)	20 (12.82)	18 (40.00)	5 (31.25)	< 0.001
Hypertensive (%)	29 (18.58)	16 (35.55)	8 (50.00)	0.003
***Biochemical parameters***				
Serum glucose (mg/dL)	89.0 (81.0–99.0)	106.0 (92.0-121.0)	103.0 (92.5-120.5)	< 0.001
Serum uric acid (mg/dL)	5.9 (5.2–6.9)	6.1 (4.8–6.6)	5.7 (4.8-6.0)	*NS*
Serum creatinine (mg/dL)	0.84 (0.71–0.92)	0.81 (0.70–0.93)	0.78 (0.68–0.89)	*NS*
eGFR (mL/min/1.73m^2^)	106 (99–114)	95 (88–101)	94 (85–99)	< 0.001
Serum total cholesterol (mg/dL)	199 (172–231)	204 (177–247)	186 (161–221)	*NS*
HDL-c (mg/dL)	48 (41–56)	47 (42–57)	45 (35–69)	*NS*
Serum triglycerides (mg/dL)	133.0 (81.0-177.0)	133.0 (102.0-188.5)	135.0 (90.0-192.0)	*NS*
AST (U/L)	35.0 (27.0-44.5)	39.0 (31.0–54.0)	60.5 (45.5–89.0)	< 0.001
ALT (U/L)	70.0 (51.0–97.0)	51.0 (41.0–78.0)	74.5 (42.5–104.0)	*NS*
Serum total bilirubin (mg/dL)	0.62 (0.46–0.86)	0.69 (0.46–0.89)	0.66 (0.51–1.15)	*NS*
Serum direct bilirubin (mg/dL)	0.20 (0.14–0.30)	0.19 (0.14–0.33)	0.26 (0.17–0.31)	*NS*
Serum albumin (g/dL)	4.7 (4.5–4.9)	4.6 (4.4–4.8)	4.5 (4.2–4.8)	*NS*
Platelet count (10^3^/µL)	239.0 (209.5–271.0)	174.0 (145.0-209.0)	138.0 (114.0-150.5)	< 0.001
***Anthropometric parameters***				
Weight (Kg)	81.7 (70.7–89.2)	82.0 (74.0–88.0)	84.1 (64.0-88.5)	*NS*
Height (cm)	170 (161–175)	163 (160–172)	166 (153–170)	0.013
BMI (Kg/m^2^)	28.2 (25.9–32.2)	29.7 (27.4–31.8)	30.1 (26.1–32.1)	*NS*
WC (cm)	99.00 (92.25–107.00)	102.00 (97.00-108.00)	106.50 (93.50-110.50)	*NS*
BRI	5.29 (4.19–6.30)	6.03 (4.85–6.51)	6.35 (5.28–7.16)	0.009
ABSI	0.081 (0.079–0.084)	0.083 (0.080–0.086)	0.085 (0.083–0.087)	0.002
***Liver scores***				
FIB-4	0.76 (0.59–0.92)	1.79 (1.52–2.10)	3.10 (2.90–3.90)	< 0.001
FIB-4 > 1.3	0 (0)	45 (100.00)	16 (100.00)	< 0.001
BLEP ≥ 2	110 (70.51)	31 (68.88)	10 (62.50)	*NS*

FIB-4: Fibrosis 4 index score; eGFR: estimated Glomerular Filtration Rate; HDL-c: High Density Lipoprotein Cholesterol; AST: Aspartate aminotransferase; ALT: Alanine aminotransferase; BMI: Body Mass Index; WC: Waist Circumference; BRI: Body Roundness Index; ABSI: A Body Shape Index; BLEP: Bright liver echo pattern; NS: *p* > 0.05

**Fig. 1:**
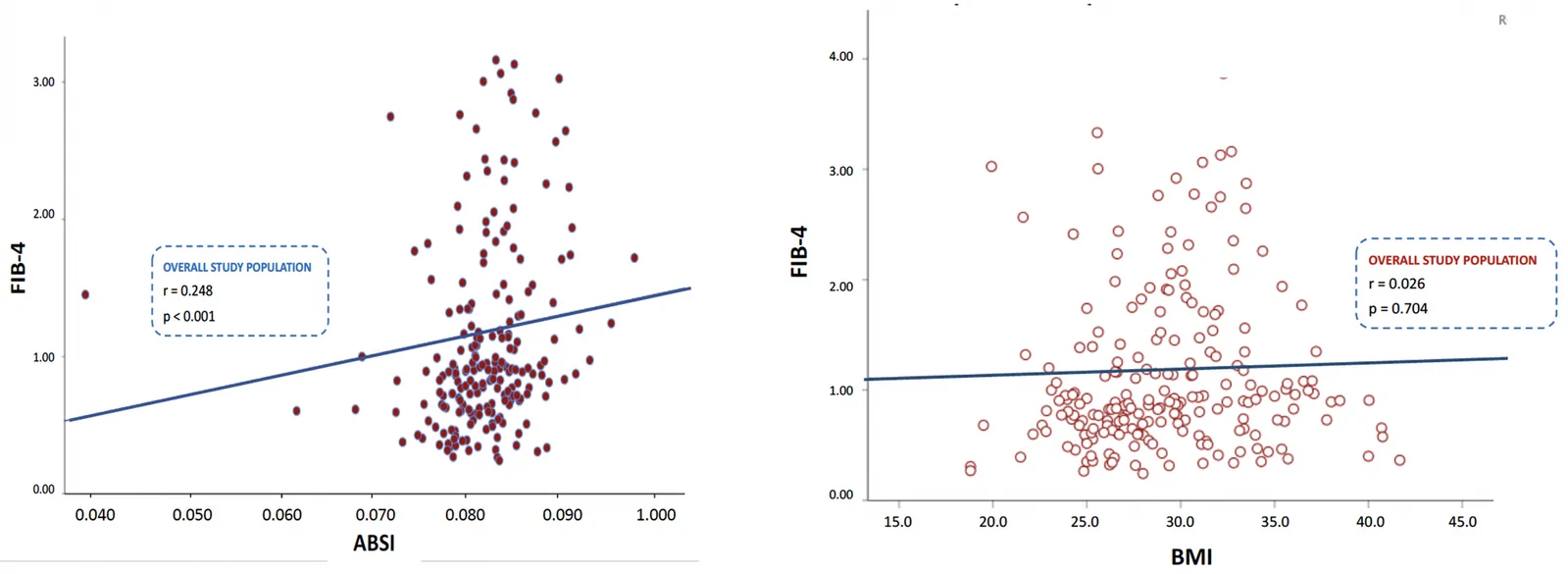
Relationship between Fibrosis-4 index (FIB-4) with A Body Shape Index (ABSI) (left panel) and body mass index (BMI) (right panel) in the overall study population

The main correlations between anthropometric indices and clinical and biochemical variables, including FIB-4, in the overall population are summarized in Table 3. BMI and WC were significantly correlated with BRI and metabolic parameters, particularly fasting glucose and triglycerides, and were inversely correlated with HDL-cholesterol. However, neither BMI nor WC was correlated with ABSI. As shown in Fig. 1, ABSI was positively and significantly correlated with FIB-4 in the overall study population (*r* = 0.248; *p* < 0.001). In contrast, no significant correlation was observed between BMI and FIB-4 (*r* = 0.026; *p* = 0.704), and the difference between slopes of the corresponding regression lines was statistically significant (*p* = 0.017).

**Table 3:** Correlations of traditional and novel anthropometric indices of adiposity clinical and biochemical variables in the overall study population

	BMI	WC	BRI	ABSI
Age (years)	0.134^*^	0.153^*^	0.357^***^	0.246^***^
Serum glucose (mg/dL)	0.265^***^	0.241^***^	0.252^***^	0.034 ^*NS*^
Serum uric acid (mg/dL)	0.131^*NS*^	0.249^**^	0.0740 ^*NS*^	0.080 ^*NS*^
Serum creatinine (mg/dL)	-0.057 ^*NS*^	0.007 ^*NS*^	-0.251^***^	-0.157^*^
eGFR (mL/min/1.73m^2^)	-0.080 ^*NS*^	-0.084 ^*NS*^	-0.157^*^	-0.069 ^*NS*^
Serum total cholesterol (mg/dL)	-0.038 ^*NS*^	0.004 ^*NS*^	0.081 ^*NS*^	0.134^*^
HDL-c (mg/dL)	-0.207^**^	-0.157^*^	0.010 ^*NS*^	0.181^**^
Serum triglycerides (mg/dL)	0.175^*^	0.140^*^	0.109 ^*NS*^	-0.031 ^*NS*^
AST (U/L)	-0.075 ^*NS*^	-0.018 ^*NS*^	0.010 ^*NS*^	0.119 ^*NS*^
ALT (U/L)	-0.022 ^*NS*^	0.020 ^*NS*^	-0.071 ^*NS*^	0.011 ^*NS*^
Serum total bilirubin (mg/dL)	-0.096 ^*NS*^	-0.076 ^*NS*^	-0.181^**^	-0.099 ^*NS*^
Serum direct bilirubin (mg/dL)	-0.103 ^*NS*^	-0.109 ^*NS*^	-0.162 ^*NS*^	-0.120 ^*NS*^
Serum albumin (g/dL)	0.029 ^*NS*^	0.008 ^*NS*^	0.014 ^*NS*^	-0.019 ^*NS*^
Platelet count (10^3^/µL)	-0.089 ^*NS*^	-0.197^**^	-0.148^*^	-0.171^*^
Weight (Kg)	0.761^***^	0.735^***^	0.402^***^	-0.109 ^*NS*^
Height (cm)	-0.110 ^*NS*^	0.117 ^*NS*^	-0.393^***^	-0.077 ^*NS*^
BMI (Kg/m2)	/	0.788^***^	0.792^***^	-0.075 ^*NS*^
WC (cm)	0.788^***^	/	0.858^***^	0.496^***^
BRI	0.792^***^	0.858^***^	/	0.475^***^
ABSI	-0.075 ^*NS*^	0.496^***^	0.475^***^	/
FIB-4	0.026 ^*NS*^	0.114 ^*NS*^	0.208^**^	0.248^***^

eGFR: estimated Glomerular Filtration Rate; HDL-c: High Density Lipoprotein Cholesterol; AST: Aspartate amino-transferase; ALT: Alanine aminotransferase; BMI: Body Mass Index; WC: Waist Circumference; BRI: Body Roundness Index; ABSI: A Body Shape Index; FIB-4: Fibrosis 4 index score

NS: *p* > 0.05; *: *p* < 0.05; **: *p* < 0.01; ***: *p* < 0.001

In the multivariable linear regression analysis performed in the entire cohort, with FIB-4 as a continuous dependent variable, age emerged as the strongest predictor (β = 0.650; *p* < 0.001), reflecting its intrinsic role in the FIB-4 calculation. ABSI remained significantly associated with FIB-4 after adjustment for covariates included in the model (β = 0.145; *p* = 0.017). In contrast, BMI – or BRI in an alternative model – was not independently associated with FIB-4 when entered in place of ABSI (Table 4a). Similarly, in the multivariable logistic regression analysis with moderate-to-severe hepatic steatosis (BLEP ≥ 2) as the dependent variable, BMI was independently associated with the outcome (OR 1.19; 95% CI 1.05–1.35; *p* = 0.006), together with fasting glucose (Table 4b). By contrast, ABSI – or BRI in an alternative model – was not independently associated with BLEP ≥ 2 when entered the model in place of BMI.

**Table 4:** Multivariable regression analyses of anthropometric indices in relation to liver fibrosis risk and hepatic steatosis severity. (A) Multivariable linear regression analysis with FIB-4 as the dependent variable. (B) Multivariable logistic regression analysis with moderate-to-severe hepatic steatosis (BLEP ≥ 2) as the dependent variable

[A] Dependent variable: FIB-4	Regression Coefficients			t	*p*
	Non-Standardized		Standardized		
	B	SE	β		
Model (R^2^ = 0.467)					
Constant	-2.362	0.684	/	-3.455	0.001
Age	0.042	0.004	0.65	10.841	< 0.001
ABSI	2.032	0.843	0.145	2.409	0.017

[B] Dependent variable: BLEP ≥ 2	Regression Coefficients		Wald	*p*-value	OR	95% CI
	Non-Standardized					
	B	SE				
Model (R^2^ = 0.239)						
Constant	-10.151	3.762	7.279	0.007	*< 0.001*	/
BMI	0.175	0.064	7.517	0.006	1.19	1.05–1.35
Serum glucose	0.035	0.014	5.87	0.015	1.04	1.01–1.07
*eGFR*	*0.03*	*0.019*	*2.438*	*0.118*	1.03	0.99–1.07
*Age*	*-0.027*	*0.025*	*1.169*	*0.28*	0.97	0.93–1.02
*Serum uric acid*	*0.18*	*0.191*	*0.885*	*0.347*	1.2	0.82–1.74
*Serum total cholesterol*	*< * *0.001*	*0.005*	*0.011*	*0.915*	1	0.99–1.01
*Serum triglycerides*	*-0.001*	*0.003*	*0.062*	*0.803*	1	0.99–1.01

FIB-4: Fibrosis 4 index score; SE: Standard Error; ABSI: A Body Shape Index

BLEP: Bright Liver Echo Pattern; SE: Standard Error; BMI: Body Mass Index; eGFR: estimated Glomerular Filtration Rate; OR: Odds Ratio; CI: Confidence Interval

**Fig. 2:**
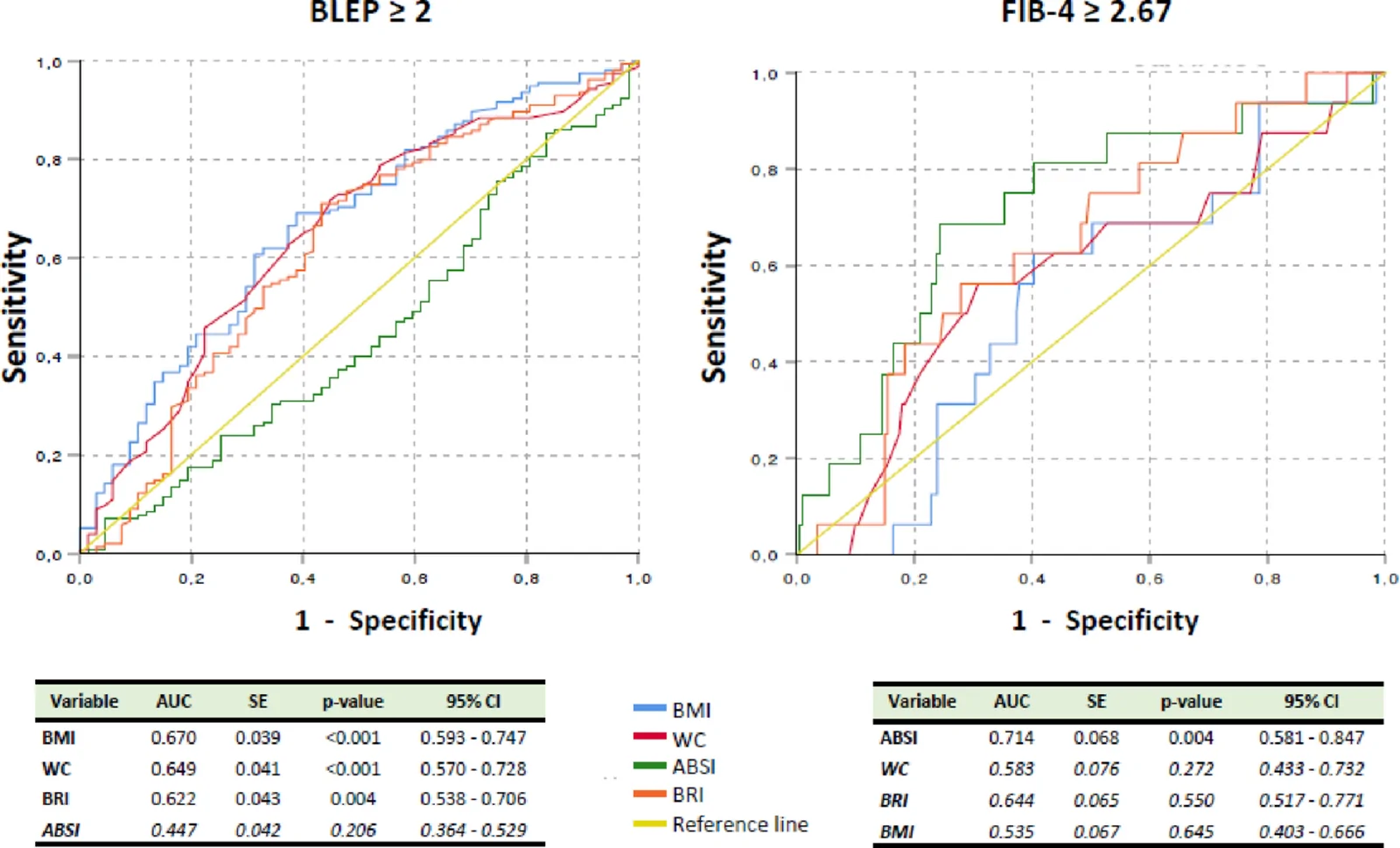
Receiver operating characteristic (ROC) curves of anthropometric indices for the discrimination of moderate-to-severe hepatic steatosis (BLEP ≥ 2, left panel) and advanced fibrosis risk (FIB-4 ≥ 2.67, right panel)

To assess the ability of each adiposity index to discriminate individuals with BLEP ≥ 2 and FIB-4 ≥ 2.67, receiver operating characteristic (ROC) curve analyses were performed. As shown in Fig. 2, BMI exhibited the highest discriminative performance for moderate-to-severe steatosis, followed by WC and BRI, whereas ABSI did not show significant discriminative performance for this outcome. By contrast, ABSI showed the highest diagnostic accuracy in discriminating advanced liver fibrosis (FIB-4 ≥ 2.67), with an AUC of 0.714 (*p* = 0.004), while BMI and WC were not discriminatory.

## Discussion

In this study, we observed that different anthropometric indices are differentially associated with features across the MASLD spectrum. While traditional adiposity indices are primarily associated with the hepatic steatosis, ABSI emerges as the main anthropometric determinant of hepatic fibrosis risk. These findings suggest that total adiposity and fat distribution may exert distinct biological effects across different phases of MASLD [29, 30].

In our study, BMI and WC were strongly and independently associated with both the presence and severity of hepatic steatosis. This is consistent with known pathophysiological mechanisms, whereby increased adiposity is linked to enhanced free fatty acid delivery to the liver and insulin resistance–mediated triglyceride accumulation within hepatocytes [7, 31].

BMI is a well-established marker of total body adiposity, reflecting overall fat and body mass rather than fat distribution [12, 14, 29]. Epidemiological and imaging studies have shown that BMI correlates with hepatic steatosis, particularly at higher ultrasonographic grades, likely through mechanisms involving insulin resistance and increased substrate availability to the liver [7, 32]. In support of this pathophysiological link, fasting plasma glucose was independently associated with steatosis severity in our population, further reinforcing the central role of metabolic dysfunction and insulin resistance in the development and progression of steatotic liver disease [33].

In contrast, BMI and WC showed no association with hepatic fibrosis risk. Instead, ABSI emerged as the only anthropometric index independently associated with elevated FIB-4 values, and this association persisted after adjustment for BMI. Notably, although the association between ABSI and FIB-4 remained statistically significant after adjustment, age was the strongest predictor of FIB-4 in our models, reflecting its intrinsic role in the score and potentially influencing the observed associations. Overall, these findings indicate a divergence from the well-established role of traditional adiposity indices in hepatic steatosis and suggest that fibrosis progression in MASLD is driven less to total adipose mass and more by adverse fat distribution and its associated metabolic and inflammatory activity [7, 19].

Previous studies have shown that ABSI reflects visceral adipose tissue distribution and preferentially identifies metabolically unfavorable fat accumulation compared with traditional adiposity indices [12, 13, 34]. Because ABSI incorporates WC relative to height and weight, it may identify individuals with disproportionate central adiposity. This phenotype may also reflect reduced lean mass and thus act as a surrogate marker of adverse body composition, consistent with sarcopenic obesity [35]. This interpretation is supported by growing evidence linking sarcopenia and reduced skeletal muscle mass to greater liver disease severity, including hepatic fibrosis, independently of BMI. Loss of muscle may mass exacerbate metabolic dysfunction through impaired peripheral glucose uptake, worsening systemic insulin resistance, and reduced secretion of anti-inflammatory myokines [36, 37].

Consistently, ABSI has been more strongly associated with cardiovascular events and mortality than BMI or WC [13]. Our findings extend these observations to liver disease, suggesting that disproportionate visceral adiposity may define a phenotype associated with fibrotic remodeling, in line with evidence linking visceral and perivisceral fat to inflammatory and profibrotic pathways [35]. Notably, FIB-4 integrates age, aminotransferase levels, and platelet count, and this finding supports the concept that hepatic fibrosis reflects the cumulative impact of chronic metabolic and inflammatory burden rather than a direct consequence of excess body weight per se [37, 38]. Importantly, the strong contribution of age to the score may partially drive the observed associations with anthropometric indices, particularly those reflecting body composition changes that occur with aging.

An alternative interpretation of our findings should also be considered. Beyond serving as a downstream marker of visceral adiposity, ABSI may capture metabolic and inflammatory perturbations arising from hepatic steatosis itself. Although hepatic fat accumulation has traditionally been regarded as a consequence of insulin resistance and visceral adiposity [31], emerging evidence suggests that steatosis may actively exacerbate metabolic dysfunction through hepatocellular lipid overload, impaired insulin signaling, and the release of pro-inflammatory mediators [7, 36]. These processes may further amplify systemic insulin resistance and contribute to adverse adipose tissue redistribution, potentially reflected by variations in ABSI [33, 37].

In this framework, ABSI could be interpreted not only as a marker of pathogenic fat distribution but also as a phenotypic indicator of metabolic and inflammatory damage originating from steatotic liver disease [37, 39, 40].

Several limitations of the present study should be acknowledged. First, the cross-sectional design precludes any inference on temporal directionality between adiposity indices, hepatic steatosis, and fibrosis risk. Therefore, the observed relationships should be interpreted as associative only. Second, hepatic steatosis was assessed by conventional B mode ultrasonography using the bright liver echo pattern, which, although widely used in clinical practice, is a semi-quantitative and operator-dependent technique and lacks sensitivity for mild steatosis. Most importantly, liver fibrosis was not assessed by histology, which remains the reference standard for fibrosis staging. Instead, fibrosis risk was estimated using the FIB 4 index, a validated non-invasive tool with high negative predictive value but limited accuracy for fibrosis quantification and staging. Accordingly, our findings should be interpreted as reflecting fibrosis risk rather than histologically confirmed fibrosis. Third, the number of patients classified as high risk for advanced fibrosis (FIB-4 ≥ 2.67) was relatively small in our cohort. This may limit the statistical robustness of analyses based on categorical fibrosis risk stratification and increases the risk of model instability. To partially address this limitation, we complemented categorical analyses with models using FIB-4 as a continuous variable, thereby leveraging the full variability of the dataset and reducing the impact of small subgroup sizes. Nevertheless, these findings should be interpreted with caution and considered primarily exploratory and hypothesis-generating. Fourth, although age was included in multivariable models, residual confounding cannot be excluded, particularly given the central role of age in the FIB-4 calculation. This may have influenced the observed association between ABSI and FIB-4. Furthermore, body composition was assessed using anthropometric indices rather than direct imaging techniques such as computed tomography, magnetic resonance imaging, or dual-energy X-ray absorptiometry, which would allow a more precise quantification of visceral adiposity and skeletal muscle mass. However, the use of simple anthropometric measures enhances the clinical applicability and scalability of our findings. Finally, the study population consisted exclusively of Caucasian individuals from a single center, which may limit the generalizability of the results to other ethnic groups or healthcare settings.

Despite these limitations, the observed associations are compatible with a potential bidirectional interplay between hepatic steatosis and fibrosis, metabolic dysfunction, and adipose tissue redistribution along the MASLD spectrum; however, this hypothesis cannot be tested within the present cross-sectional framework and longitudinal studies are needed. Further studies including liver stiffness measurement (e.g., transient elastography), histological assessment, as well as prospective and multicenter validation cohorts, are warranted to validate and extend the present observations, as well as to identify the clinical utility of shape-based anthropometric indices in the risk stratification of MASLD.

## Conclusion

In our study, traditional adiposity indices were primarily associated with the presence and severity of hepatic steatosis, whereas ABSI emerged as the only anthropometric index independently associated with elevated FIB-4 values. However, given that FIB-4 is a composite score and that the number of patients at high fibrosis risk was limited, this finding should be interpreted cautiously and regarded as indicative of an association with fibrosis risk rather than with confirmed fibrosis. Overall, these findings suggest that fibrotic risk in MASLD may be fully explained by total body adiposity alone but may also be related to adverse visceral fat distribution. The combined use of traditional and shape–based adiposity indices may improve risk stratification and support more personalized clinical management in MASLD.

## Data Availability

The datasets generated and/or analyzed during the current study are not publicly available but are available from the research team at the center where the study was conducted upon reasonable request.
